# An Augmented Reality System Using Improved-Iterative Closest Point Algorithm for On-Patient Medical Image Visualization

**DOI:** 10.3390/s18082505

**Published:** 2018-08-01

**Authors:** Ming-Long Wu, Jong-Chih Chien, Chieh-Tsai Wu, Jiann-Der Lee

**Affiliations:** 1 Department of Electrical Engineering, Chang Gung University, Taoyuan 333, Taiwan; elmowuming@gmail.com; 2 Degree Program of Digital Space and Product Design, Kainan University, Taoyuan 333, Taiwan; jongchih@gmail.com; 3 Department of Neurosurgery, Chang Gung Memorial Hospital, LinKou, Taoyuan 333, Taiwan; woodie2@adm.cgmh.org.tw; 4 Department of Electrical Engineering, Ming Chi University of Technology, New Taipei City 24301, Taiwan

**Keywords:** image alignment, improved-ICP algorithm, head-mounted display

## Abstract

In many surgery assistance systems, cumbersome equipment or complicated algorithms are often introduced to build the whole system. To build a system without cumbersome equipment or complicated algorithms, and to provide physicians the ability to observe the location of the lesion in the course of surgery, an augmented reality approach using an improved alignment method to image-guided surgery (IGS) is proposed. The system uses RGB-Depth sensor in conjunction with the Point Cloud Library (PCL) to build and establish the patient’s head surface information, and, through the use of the improved alignment algorithm proposed in this study, the preoperative medical imaging information obtained can be placed in the same world-coordinates system as the patient’s head surface information. The traditional alignment method, Iterative Closest Point (ICP), has the disadvantage that an ill-chosen starting position will result only in a locally optimal solution. The proposed improved para-alignment algorithm, named improved-ICP (I-ICP), uses a stochastic perturbation technique to escape from locally optimal solutions and reach the globally optimal solution. After the alignment, the results will be merged and displayed using Microsoft’s HoloLens Head-Mounted Display (HMD), and allows the surgeon to view the patient’s head at the same time as the patient’s medical images. In this study, experiments were performed using spatial reference points with known positions. The experimental results show that the proposed improved alignment algorithm has errors bounded within 3 mm, which is highly accurate.

## 1. Introduction

With the rapid development of computer and image processing technologies, Augmented Reality (AR) has become more widely used in many different areas, such as education [1], entertainment [2], medicine [3], etc., and it also adds the feelings of reality for the user compared to Virtual Reality (VR) [4]. In AR applications, images of the real world are captured with a camera and virtual objects are drawn on top of them according in the user’s designated locations. This can be useful in medical applications such as Image-Guided Surgery (IGS). Nowadays, the use of IGS system [5] plays a very significant role in the healthcare industry. The motivation for introducing IGS is to reduce invasiveness and to implement non-contact alignment. IGS will improve surgical safety and help to avoid contact with the patient with the physical alignment instrument, which would pose a risk of infection. In 2015, Kersten-Oertel et al. [6] applied augmented reality to Image-Guided Neurosurgery System (IGNS) and used expensive commercial spatial alignment instruments that require physical contacts, which our proposed system seeks to avoid. In 2014, Deng et al. [7] proposed using a tablet computer to achieve image-guided neurosurgery. However, his proposal included the use of multiple markers which need to be placed on the tablet computer and by the dummy head to correctly align and display the head on the tablet computer, so it is not actually superimposing the medical image on top of the skull’s surface. In this study, the preoperative medical imaging data obtained of the patient would help the medical staff to evaluate the lesion and to confirm the location of the lesion using AR. The preoperative surgical image-assisted navigation system can also be used to plan the surgery routine and help locate the tumor. Various studies such as by Macedo et al. [8] intensified the research in the use of medical imaging in augmented reality. In other words, in the use of AR in surgery, the patients’ medical images must be obtained using various instruments before the operation, according to the needs of the surgeries and the conditions of the patients, medical images such as Computed Tomography (CT), Magnetic Resonance Imaging (MRI) and Ultrasound Image can be used to analyze the location of the lesion through these preoperative data [9].

Today’s surgical medical imaging technology still requires surgeons to look up from time to time to the images on the monitor to aid the progress of the surgery [10]. Microsoft has developed several Hololens applications for medical treatment [11,12], but most of them have not been applied to the alignment of the virtual images with the human body; instead, the virtual objects are placed in an empty space environment for educational guidance purposes. However, if it is possible to display the virtual data derived from medical images at the same time as the patient [13], then this technology is bound to be the trend of the future. This goal requires accurate alignment of the positions of the virtual data with the patients.

Thus, how to correctly align medical images with patients’ positions is very crucial. The traditional alignment method, the Iteration Closet Point (ICP) algorithm, which was introduced by Besl and McKay [14], and Zhang [15], uses rigid transformation and continuous iterative calculation, to find the best coordinate transformation for a group of 3D-points from one coordinate system to another coordinate system via translations and rotations [16]. In 2001, Penney et al. [17] proposed the Stochast-ICP algorithm, which uses a random perturbation at each iteration to escape from the local minimum, and to converge on the globally best solution. However, their results show that the total number of iterations will become much larger than the traditional ICP method and would be time-consuming. In 2010, Andriy et al. [18] proposed the Coherent Point Drift (CPD) alignment algorithm. Its main idea is to initialize a Gaussian Mixture Model (GMM) and use the Estimation Maximization (EM) calculation to move the centroid of the mixture as a whole. This algorithm has three versions, which are rigid, affine, and non-rigid methods. The rigid method requires that the rotation matrix must be an orthogonal and positive definite matrix. The affine method does not require the rotation matrix to be an orthogonal and positive definite matrix, but it does not consider the scaling factor, so there would still be a small possibility of cloud deformation. The above mentioned ICP-based alignment methods, including the traditional ICP, Stochastic-ICP, and CPD-ICP, all must have a good initial position to converge to desired solutions. In addition, in 2015, Yang et al. [19] proposed the Go-ICP (Globally Optimal-ICP) algorithm, where rigid Euclidean alignment are performed for two sets of points in the L^2^ space under the amount of error as defined by the ICP. This algorithm incorporates a Branch and Bound (BnB) method that effectively searches in the 3D motion space SE(3), and, by using the special structure of the SE(3) geometry, the upper and lower limits of the alignment error function can be deduced. Because of this, the authors claimed that a good initial location is not required for the algorithm to get the best global solution. This claim is examined in the Experimental Results Section 3.

## 2. Method

### 2.1. Modeling from Medical Images

**Figure 1:**
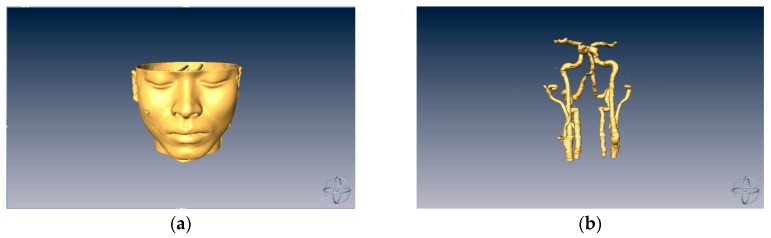
(**a**) A dummy head model; and (**b**) model of intracranial vascular tissue.

In our experiment, a dummy model was used to simulate the head of a patient. First, a CT scan of the dummy model was performed and the resulting stack of images were processed using image processing methods [20] in order to capture the regions of interest (ROI) and a threshold was set on the image stack to obtain the 3D modeling data, as shown in Figure 1.

### 2.2. Surface Data Alignment

Currently, the most widely used surface alignment algorithm for three-dimensional objects is the Point Cloud Library-Iteration Closest Point (PCL-ICP) algorithm [21]. This algorithm aligns two sets of point cloud data and its processes are divided into four main steps: (1) sample from the original point cloud data; (2) determine the initial correspondence point data; (3) remove the erroneous corresponding points; and (4) derive the coordinate transformation. First, it assumes two corresponding point sets P and Q, or reference and floating, are determined, and the number of corresponding points is *N*. The optimal coordinate transformation between P and Q is iteratively calculated by the least square method to obtain a rotation matrix R and a displacement matrix T, set in the same coordinate system. The main method aligns the floating point group to the reference point group by means of iteration.

In this study, a RGB-D sensor [22] and Point Cloud Library (PCL) [23] are used to reconstruct 3D information of the surface of real objects. The traditional ICP alignment algorithm is not considered for our system. It is because there are two distinct disadvantages in the traditional ICP algorithm: The first disadvantage is that an arbitrarily different starting position may cause the algorithm to ends up in a locally optimal solution instead of the globally optimal solution. The second disadvantage is that grossly different points or noise in the non-overlapped area may affect the result of the alignment. Thus, to solve these two problems, a perturbation mechanism is added in each iteration to facilitate escaping from locally optimum solutions. For further improvements, the improved alignment algorithm, i.e., I-ICP, adds the concept of weights into the strategy: According to the distance between the two points of the correspondence in each group, different weight values are assigned to each point to reduce the error caused by the matching wrong points. This weighting scheme is so designed because, when a point in the floating point group searches for its corresponding point in the reference point group, it may be possible that multiple floating points find correspondences in the same reference point. Therefore, the weighting values are assigned to the floating point group to prevent the accumulation of multiple alignment errors. The median value is used in order to avoid choosing the extreme values thus avoiding misalignments. 

**Figure 2:**
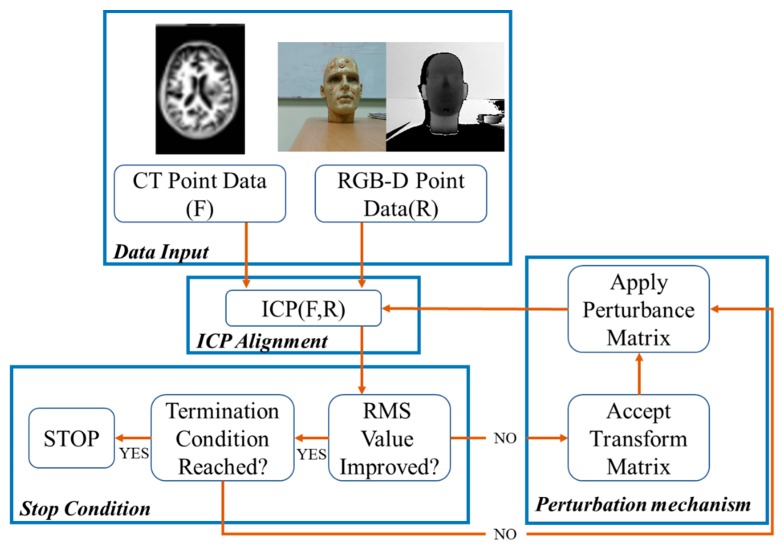
The flowchart of I-ICP alignment algorithm.

The procedure of the I-ICP alignment algorithm can be divided into four parts: data input, ICP registration, the stop condition and the perturbation mechanism. The flowchart of the algorithm is shown in Figure 2.

The I-ICP Procedure

• Step 1: Data Input

Assuming that the surface of the preoperative CT image is taken as a set of reference points *R*, then $R=\left\{r_{j}\left(x,y,z\right),1\leq j\leq N_{R}\right\}$, and the facial feature points captured by the RGB-D senor and calculated with the PCL library and the KAZE [24] algorithm are to be taken as a set of floating points *F*, then $F=\left\{f_{i}\left(x,y,z\right),1\leq i\leq N_{f}\right\}$.

• Step 2: ICP Alignment

For any given point *f* in the set floating data set *F*, find its nearest point $r_{j}$ in the reference data set *R*, by calculating the minimum distance between *f* and each $r_{j}$, as shown in Equation (1):

(1) $$d\left(f,R\right)=\min_{j\in \left(1,\ldots N_{R}\right)}d\left(f,r_{j}\right)$$

where *d* is the minimum distance calculated by the corresponding points, and *median* is the middle number in each group after the corresponding points are sorted from the smallest to the largest as in Equation (2):

(2) $$median\left(d_{j}\right)=\{\begin{array}{c} d_{j=\frac{N_{F}}{2}}\\ d_{j=\frac{N_{F}+1}{2}} \end{array}\;if\;\begin{array}{c} N_{F}\;is\;even\\ N_{F}\;is\;odd \end{array}$$

Then, according to the distance calculated by each corresponding point, different weight values are given in Equation (3):

(3) $$\delta =\{\begin{array}{c} 1\\ \frac{madian}{d} \end{array}\begin{array}{c} \;if\;d<median\\ otherwise \end{array}$$

where $j=\left\{d_{1},d_{2},d_{3},\ldots .d_{N_{F}}|d_{1}<d_{2}<d_{3}\ldots \mathrm{etc}.\right\}$, and *N_F_* is the total number of points in the set *F*. After the assignment of the weight values of the corresponding points in each group is completed, an objective function, *RMS*, is calculated as in Equation (4):

(4) $$RMS=\sqrt{\frac{1}{N_{F}}\overset{N_{F}}{\underset{i=1}{\sum }}\delta_{i}\cdot d_{i}}$$

A set of transformation matrix T′ is obtained by the ICP calculation, and the last calculated *RMS* is used as the evaluation value [25]. If this value is less than the evaluation value calculated in the previous recursion, then T′ is used to replace the currently optimal transformation matrix T, and the disturbance mechanism stage, Step 4, is entered. Otherwise, it should be determined whether the stop condition has been reached in Step 3.

• Step 3: Stop Condition

If the *RMS* value is less than the pre-set threshold or the number of recursions, *k*, is greater than the pre-set maximum number of recursions, then the execution stops and ends.

• Step 4: Perturbation Mechanism

**Figure 3:**
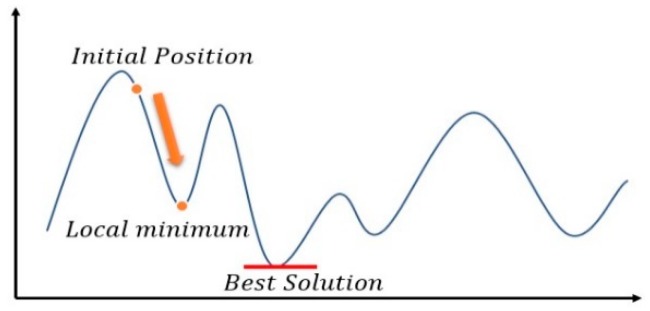
ICP Trapped in Locally Optimal Solution.

The ICP method uses the value calculated from the objective function as the evaluation value in each recursion to obtain the minimum evaluation value. Therefore, in each iteration, the calculated evaluation value would be smaller than that estimated in the previous recursion. This type of evaluation method is similar to the gradient descent method shown in Figure 3.

**Figure 4:**
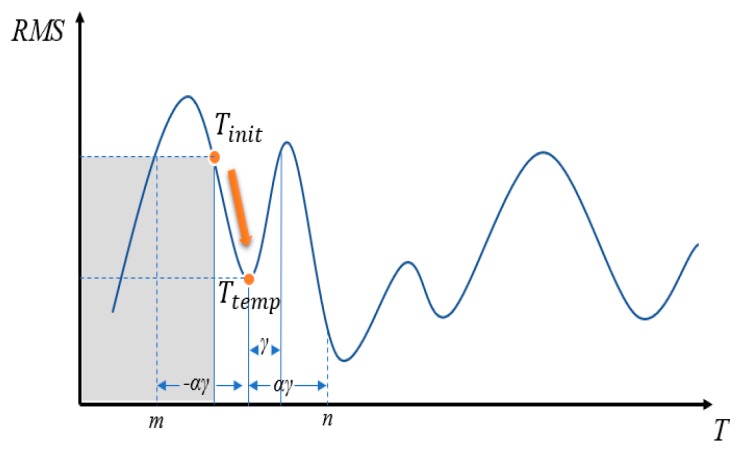
Example Illustration of the RMS Solution.

To speed up the search, the golden section search [26] is used: As illustrated in Figure 4, if the function’s interval is between −αγ and αγ, then the golden ratio *ϕ* can be chosen, as shown in Equation (5), and αγ is then set according to the ratio, as shown in Equation (6).

(5) $$\varphi =\frac{-1+\sqrt{5}}{2}\approx 0.618$$

(6) αγ=φ·(n−m)

If $RMS\left(T_{temp}\right)>RMS\left(T_{init}\right)$, then a local minimum falls between *n* = (*T_tem_*_p_ + αγ) and *T_init_*. On the other hand, if $RMS\left(T_{temp}\right)<RMS(T_{init}$), then a local minimum falls between *T_init_* and *m* = (*T_temp_* − αγ), and the gray area shown in the figure may be ignored during the search. A minimum value can be found through by searching endlessly, however, the result may not necessarily be the globally best solution, and in this case, we call the solution a local solution. To overcome this problem, we propose to use a perturbation method to make the search jump out of the local solution and restart the search for the best solution.

If we let

(7) $$\gamma =|T_{init}-T_{temp}|$$

then use the value to calculate the differences in rotation in the *X*-axis, *Y*-axis, and *Z*-axis. Given a parabolic probability function, it can be used to assign probability to a variable *y*, representing its probability of being selected, as given in Equation (8):

(8) $$p\left(y\right)=\{\begin{array}{c} \alpha y^{3}\\ 0 \end{array}\begin{array}{c} \;if\;-\alpha \gamma <y<\alpha \gamma \\ otherwise \end{array}$$

If the defined solution space is not sufficiently large, it may happen that search cannot escape from the local solution. Therefore, it is necessary to expand the scope of the solution space using a scale factor *α*, where *α* is a coefficient to expand the perturbation range until the stop condition is satisfied.

### 2.3. Detection of the Marker Board

To prepare pre-operative spatial positioning, a preset marker plate is placed next to the dummy head model during surgery to establish an unchanging relationship between the two. When the camera detects the marker plate, the virtual image is superimposed on the dummy head model.

**Figure 5:**
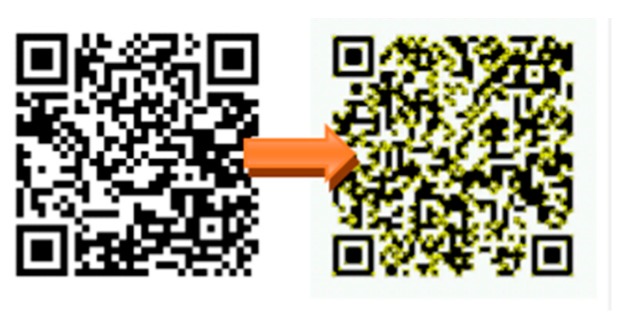
A self-made Vuforia marker.

The mixed reality system uses the Vuforia’s image tracking SDK [27], which can identify and track feature points on a 2D planar images as well as 3D images. Compared to AR Toolkit [28], Vuforia SDK can track the feature points and identify the image even if the image is partially occluded. This study uses a self-made QR code marker, shown in Figure 5, as features to be tracked.

**Figure 6:**
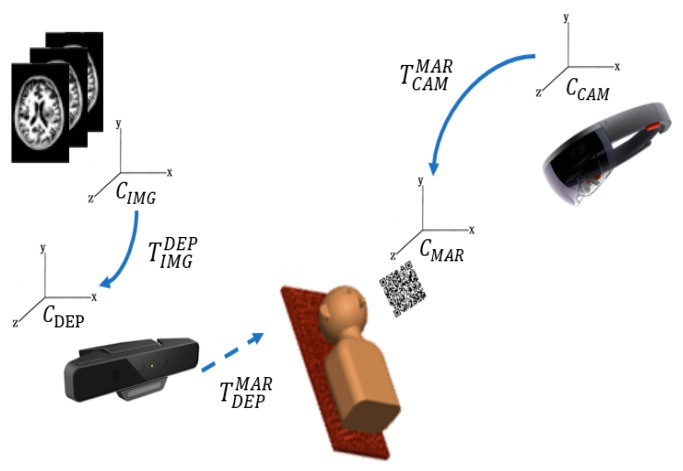
The process of conversion between different coordinate systems.

Because the HoloLens device has its own coordinate system [29], to observe pre-operative medical image overlays on the target, the coordinate systems need to be integrated first. First, the I-ICP transforms the coordinate system, $C_{IMG}$, of the medical image data to the RGB-D sensor coordinate system $C_{DEP}$, using $T_{IMG}^{DEP}$. Then when the QR-Code marker is placed in the field of the RGB-D sensor, and the conversion transformation, $T_{MAR}^{DEP}$, to the world coordinate system can then be calculated. Finally, the conversion transform, $T_{CAM}^{MAR}$, of the HoloLens’ coordinate system, $C_{CAM}$, to the QR-Code marker’s coordinate system, $C_{MAR}$, completes the conversion. This process is illustrated in Figure 6.

The hardware devices used in this study consisted of a desktop computer with Windows 10 OS, a 64-bit Intel(R) Core(TM) i5-4460 CPU, a RGB-D sensor, and Microsoft HoloLens [30]. The HoloLens system uses Windows 10 OS, includes a CPU and a GPU, and has a display resolution of 1280 × 720. In addition, a holographic processing unit (HPU) is also designed to handle spatial mapping using SLAM (Simultaneous Localization and Mapping) [31,32] to process spatial projections [33], and allows the user to access the user interface in the augmented reality environment through voice and gestures. This system was developed with Unity [34].

## 3. Experimental Results

In this section, the results of comparisons between the proposed I-ICP algorithm and other ICP-based alignment algorithms using non-medical and medical datasets are presented.

### 3.1. Alignment Tests Using a Non-Medical Dataset

**Figure 7:**
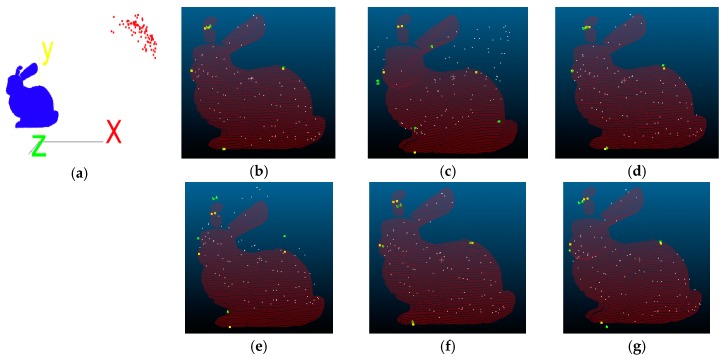
Stanford Bunny alignment results: (**a**) initial positions; (**b**) I-ICP; (**c**) PCL-ICP; (**d**) Go-ICP; (**e**) CPD-ICP (rigid); (**f**) CPD-ICP (affine); and (**g**) CC-ICP.

The Stanford Bunny [35], shared by Grek Turk and Marc Levoy in 1994, was used as test data. The number of data points of the Bunny is 40,256, so first the number data points is reduced to 100, then rotated 50 degrees along the *X*-, *Y*-, and *Z*-axis before a translation of 200 mm is performed. The processed data points are then treated as the floating point set, and ICP-based alignment tests are performed with the original data points. The visual results of the alignment tests using proposed I-ICP, PCL-ICP, Go-ICP, CPD-ICP (rigid), CPD-ICP (affine), and CloudCompare-ICP [36] are shown in Figure 7, where the red cloud points are the reference points, the yellow dot are the five marker points in the reference point cloud, the white cloud points are the floating points, and the green dots are the five markers in the floating point cloud. The Root-Mean-Square (*RMS*) error is used as an evaluation, as shown in Table 1. To achieve the demands of accuracy, five reference points are first obtained for verification of the errors, as shown in Table 2, and the unit of measurement is mm.

**Figure 8:**
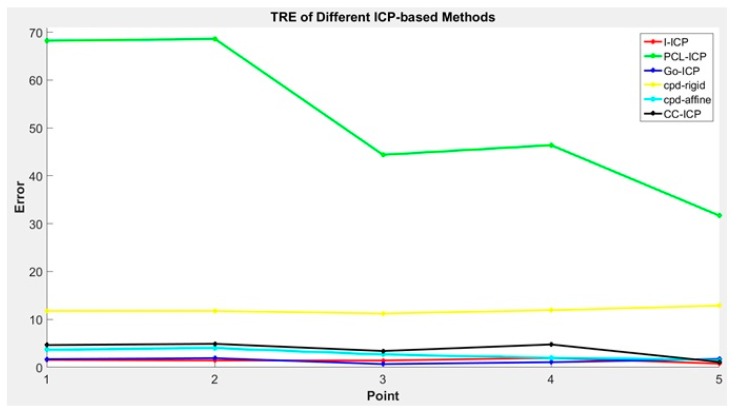
Compare the TRE of different ICP methods.

The TRE values are presented in Figure 8. In general, the Target Registration Error (TRE) [17] is a measurement standard to evaluate error in aligning image to the physical space. Assume that $p_{i}^{valid}$ is the validation point, shown in yellow in Figure 7, and $p_{i}^{f}$ is the floating point after the ICP transform, shown in green in Figure 7. Then, the TRE can be calculated using Equation (9):

(9) $$TRE_{i}=∥p_{i}^{valid}-p_{i}^{f}∥$$

Comparing the results in Table 1 and Table 2 and Figure 8, we found that the *RMS* errors of all ICP-based methods used in the comparison are very small, with CC-ICP appearing to be the best performer. However, upon comparing the average error of the reference points, we found that the results of most of the ICP-based methods, except for the proposed I-ICP and Go-ICP, are over 3 mm.

### 3.2. Dummy Head Alignment Test

**Figure 9:**
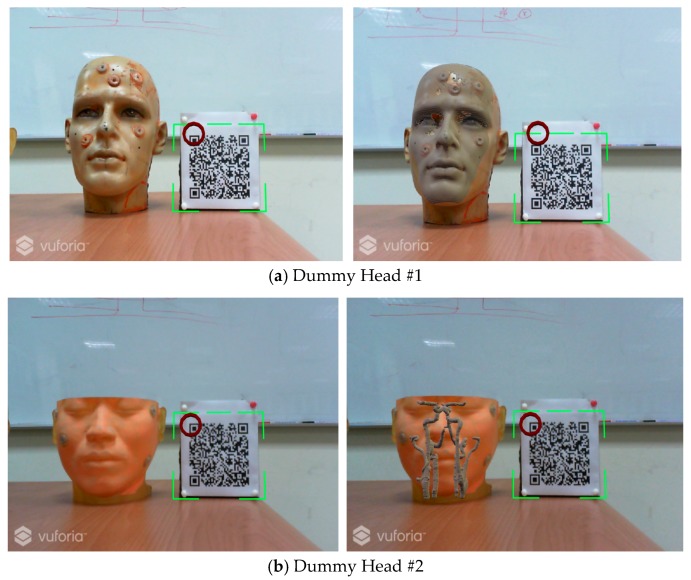
The Result of Marker Detection and Overlaying the Medical Images over the Dummy Head.

In this test, a marker plate is placed next to the dummy head while keeping their coordinate relationship unchanged, so when the HoloLens camera detects the QR Code feature point, the medical image is displayed on the screen and then overlays the dummy head in the correct position, as shown in Figure 9.

**Figure 10:**
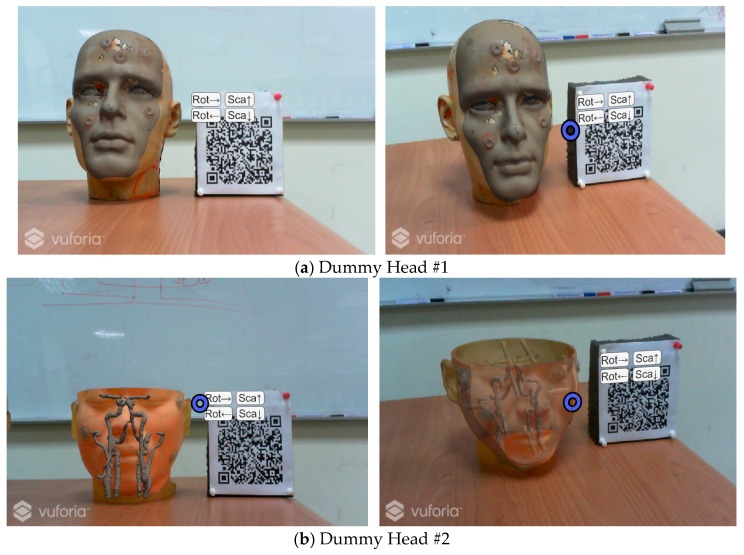
Display of cursor for interactive rotation, and scaling.

To facilitate the doctor to observe more detailed information, the design of the user interface in the augmented reality environment allows the user to change the rotation angle and the scale of the virtual object through the use of gestures [37], as shown in Figure 10.

**Figure 11:**
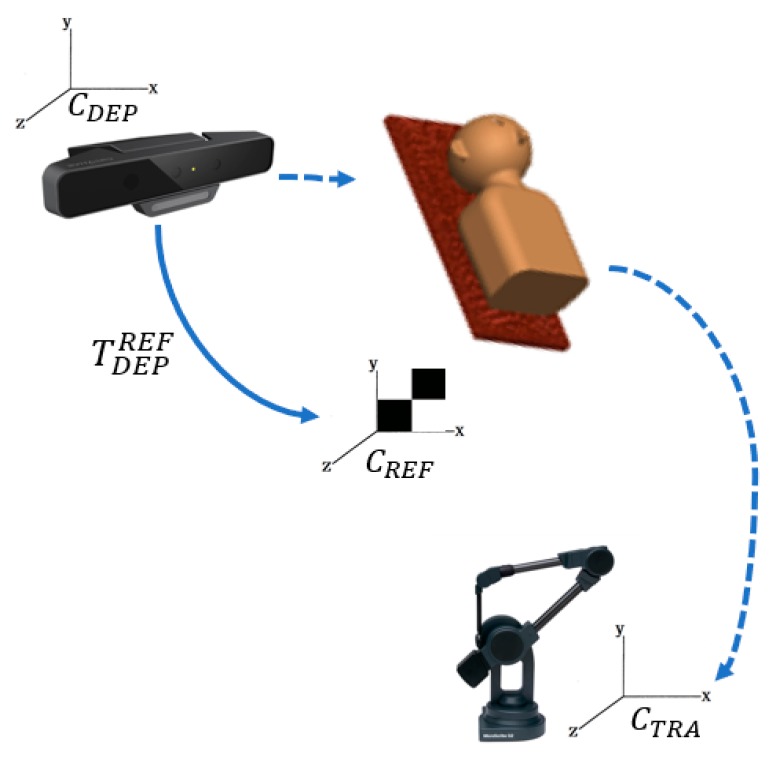
Diagram of the computation of coordinate conversion validation errors.

**Figure 12:**
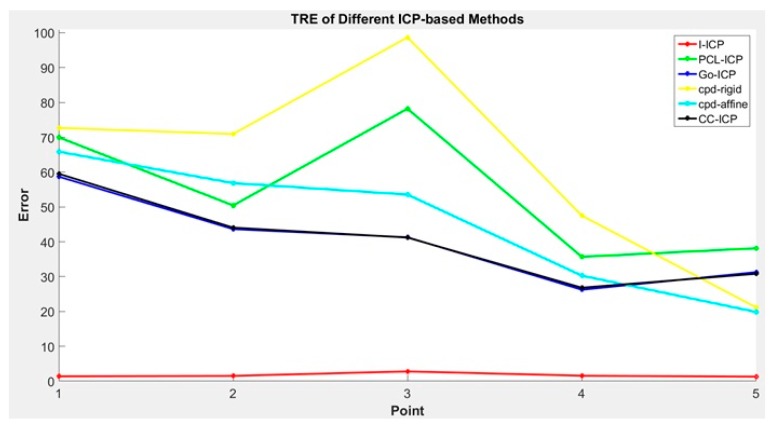
Comparison of the TREs for different ICP methods.

To compare I-ICP’s accuracy with the other ICP-based alignment algorithms, five reference points were obtained using the MicroScribe G2X Digitizer coordinate tracking device. They are used as the standard for verification of errors [38], and taken in the digitizer’s own coordinate system, $C_{TRA}$. A checkerboard is used in the reference coordinates, $C_{REF}$, to align the reference points to the world coordinate system using the transform $T_{DEP}^{REF}$. This process is illustrated in Figure 11. The experimental results are the results of taking the averages of the results of ten experiments in comparing the alignment results of I-ICP with CloudCompare-ICP, PCL-ICP, Go-ICP, CPD-ICP (rigid), and CPD-ICP (affine). The experiments were performed without using a good initial starting point, and using mm as the unit of measure for error. The time used for alignment is also displayed. The results of the comparison for Dummy Head #1 in Figure 10 is displayed in Table 3 and their TREs are shown in Figure 12. 

**Figure 13:**
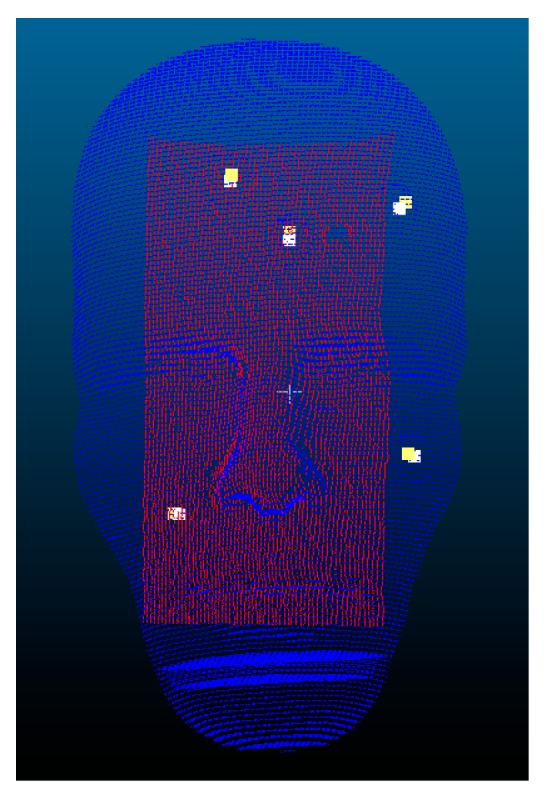
Alignment result of using I-ICP on Dummy Head #1.

The visual results of the I-ICP alignment are shown in Figure 13, where the red point cloud is the set of floating points, and the blue point cloud is the set of reference points.

The second experiment included performing an initial coarse alignment, making the floating point cloud closer to the reference point cloud. A greedy rough alignment method uses the rotation-invariant property, so it is possible to obtain only a locally optimum solution. Therefore, the Sampling Consensus Initial Alignment (SAC-IA) [39] method was used to do the coarse alignment. It first performs a Fast Point Feature Histograms (PFFH) to obtain the sampling features, then searches for their corresponding points in the reference point cloud, followed by calculating the transformation matrix between these corresponding points. The time takes for rough alignment is about 370 s for the given dataset. From the results in Table 4, it can be seen that the alignment errors for I-ICP, PCL-ICP and CPD-ICP (affine) methods are all about 3 mm. In addition, the results show that most of ICP-based methods can be improved by performing a coarse alignment first. 

## 4. Discussion

There are several requirements for many of the ICP-based alignment algorithms published in the literature [14,15,16,17,18,19] for them to converge to acceptable solutions. These requirements include that a good starting position must be given, and that the number of points in the two sets of original point clouds to be aligned must be approximately equal. To avoid these requirements, in the I-ICP algorithm, we added the golden section search, perturbations and weighting mechanisms. These mechanisms are added to increase the speed for searching for the smallest value, avoid falling into locally optimal solution during search as well as to avoid the adverse influence of outlier points, respectively. According to the results in the Stanford Bunny alignment experiment, it is found though the *RMS* values all fell within 1.0, but the reference points’ errors show that there are gaps between the PCL-ICP [21] and the CPD-ICP [18] alignment results and the actual reference points’ positions. This observation shows using the *RMS* errors alone as the measure of comparison is not sufficient to describe the accuracies of different alignment methods. This is because the *RMS* errors only describe the overall correspondence accuracy, so a small *RMS* error value would not guarantee that individual target alignment error would also be small. Therefore, TRE (Target Registration Error) is also used in addition to the *RMS* error as the final evaluation standard. 

In other words, if the *RMS* errors of two alignment methods are similarly small, then the TRE value can be referenced to differentiate their performances. We found that, although the CC-ICP [36] was faster than the I-ICP method proposed in this study, from the average TRE measurements, it is obvious that the I-ICP has better registration performance. In the experiment using the dummy head, shown in Table 3 and Table 4, we used the TRE measurements [40] to validate alignment accuracy, and five markers on the dummy’s head were used to evaluate alignment errors. The results, shown in Table 3, were the performance comparisons of using multiple ICP-based alignment methods, where a good starting position was deliberately withheld. It can be seen from the results that the GO-ICP algorithm failed to converge. This is in spite of the fact that the designers of the GO-ICP algorithm claimed that it does not need a good starting position to converge to the global solution. Our experiment showed that this claim is untrue when the two sets of cloud point data are far apart: its maximum error on the *y*-axis at the first marker point was 126.17 mm. In the same experiment, the CPD-ICP (rigid) algorithm had the greatest amount of error amongst all methods compared; its errors on the *y*-axis of marker points 1, 2, and 3 were as high as 176.52 mm. On the other hand, the proposed I-ICP can converge to the global optimal solution, with or without a good starting position. If two sets of cloud points are different in size, it would be necessary to consider a scaling factor while aligning. Because the CPD-ICP (affine) algorithm does not consider the scaling factor, its alignment result may cause the phenomenon of point cloud deformation. In that same experiment, the results showed that its registration errors on the y-axis of marker points 1, 2, and 3 were also very large, while the I-ICP algorithm proposed by this study had the best performance overall; its errors fell mostly within 3 mm, while spent only 7 s in computation time. 

This study investigated the problem of surface reconstruction by combining a RGB-D sensor and the PCL and KAZE algorithms to calculate feature points. However, the current setup may not find sufficient feature information in bright light environment and overly smooth surfaces.

## 5. Conclusions

This study proposed an augmented reality system for on-patient medical display. The patient’s CT images are taken before the surgery and then accurately positioned on the patient’s face through the use of the Improved-Iterative Closest Point alignment algorithm proposed in this study. With this system, surgeons no longer need to look up and watch the LCD as they used to do. This system can assist in operations by superimposing virtual images on real images and display the results on the head-mounted display screen. The proposed system eliminates the need for physical contact with the patient, which is one of its design goals, and thus adds many conveniences. The experimental results show that the proposed method has better efficiency and accuracy. Its average TRE measurement is less than 3 mm, and within the range acceptable by doctors, which is the consensus opinion of consulted professional neural surgeons. 

In the future, our methods can be further improved by using the structure light equipment to acquire the surface information when sufficient feature points on smooth surface cannot be extracted under normal lighting. In addition, the current setup still uses marker-based localization to complete space coordinate integration; it is expected that this will not be necessary and that Hololens itself will provide depth information to its users in the future, thus reducing the need for excessive coordinate conversions.

## Figures and Tables

**Table 1:** Comparisons of Root-Mean-Square errors.

	I-ICP	PCL-ICP	Go-ICP	CPD-ICP (Rigid)	CPD-ICP (Affine)	CC-ICP
*RMS*	0.0008	0.0008	0.0007	0.0007	0.0008	0.0003
Run time	5 s	75 s	14 s	17 s	20 s	3 s

**Table 2:** Reference points errors.

Method	Axis	Point 1	Point 2	Point 3	Point 4	Point 5	Avg.
I-ICP	*x*	2.02	2.23	−0.27	−1.05	0.38	1.19
	*y*	1.08	0.72	0.24	−0.63	1.55	0.84
	*z*	−1.51	−1.34	−3.73	−4.22	0.38	2.23
PCL-ICP	*x*	−27.68	−23.14	37.34	94.91	−51.08	46.83
	*y*	−47.67	−49.57	−58.20	37.91	26.27	43.92
	*z*	129.26	133.14	37.60	−6.40	−17.72	64.82
Go-ICP	*x*	−2.79	−2.55	−0.57	2.01	−0.99	1.78
	*y*	−0.43	−0.90	−0.59	0.82	3.82	1.31
	*z*	1.87	2.26	0.87	−0.36	−0.40	1.15
CPD-ICP (rigid)	*x*	1.65	1.84	−1.42	−2.85	−0.68	1.69
	*y*	19.60	19.25	19.85	19.16	20.66	19.70
	*z*	14.07	14.23	12.47	13.76	17.29	14.36
CPD-ICP (affine)	*x*	4.14	4.56	3.52	−0.51	−2.46	3.04
	*y*	−4.79	−5.14	−1.32	2.43	0.33	2.80
	*z*	2.14	2.35	−3.19	−3.03	1.73	2.49
CC-ICP	*x*	−5.99	−5.73	−0.57	6.65	−0.47	3.88
	*y*	−3.40	−4.03	−6.97	−5.15	2.37	4.38
	*z*	4.55	4.96	2.67	−2.54	−0.48	3.04

**Table 3:** Results of alignment errors comparison.

Registration Method	Axis	Point 1	Point 2	Point 3	Point 4	Point 5	Avg.	Time
I-ICP	*x*	2.09	0.93	−4.71	3.26	−2.18	2.63	7 s
	*y*	−1.68	−2.89	−2.84	0.25	−0.03	1.54	
	*z*	−0.37	0.73	−0.83	1.19	1.69	0.96	
PCL-ICP	*x*	23.57	15.34	6.01	−2.03	1.40	9.67	234 s
	*y*	−160.44	−125.27	−146.21	14.27	53.77	99.99	
	*z*	25.97	−10.69	−82.38	−90.77	59.30	53.82	
Go-ICP	*x*	43.25	42.40	18.98	−13.59	−2.27	24.10	176 s
	*y*	−126.17	−85.92	−100.18	55.79	77.92	89.20	
	*z*	6.80	2.60	4.73	−9.32	−13.68	7.43	
CPD-ICP (rigid)	*x*	22.37	16.68	7.12	6.84	10.16	12.63	180 s
	*y*	−176.52	−140.46	−161.69	−1.51	37.43	103.5	
	*z*	−19.17	−55.71	−127.24	−134.20	15.87	70.44	
CPD-ICP (affine)	*x*	15.53	12.78	−1.19	−14.25	−10.57	10.86	200 s
	*y*	−151.02	−125.56	−114.06	43.91	40.88	95.09	
	*z*	−31.05	−32.24	−45.41	−32.71	−8.13	29.91	
CC-ICP	*x*	42.87	41.81	17.72	−14.50	−1.79	23.74	12 s
	y	−126.21	−85.64	−99.05	56.67	77.03	88.92	
	z	9.50	4.77	6.86	−9.30	−13.65	8.81	

**Table 4:** Results of alignment errors comparison.

Registration Method	Axis	Point 1	Point 2	Point 3	Point 4	Point 5	Avg.	Time
I-ICP	*x*	3.04	2.13	−3.08	3.71	−1.31	2.65	8 s
	*y*	−1.09	−2.33	−2.36	0.78	0.68	1.45	
	*z*	1.62	2.49	0.95	1.78	2.15	1.80	
PCL-ICP	*x*	4.07	2.92	−2.37	5.23	−0.88	3.09	138 s
	*y*	−0.19	−1.43	−1.26	1.50	0.97	1.07	
	*z*	−0.16	0.99	−0.44	1.54	1.75	0.98	
CC-ICP	*x*	−3.90	−4.69	−9.75	−1.38	−8.03	5.55	36 s
	*y*	1.81	0.47	0.57	3.47	3.20	1.90	
	*z*	0.50	1.53	0.05	1.34	1.58	1.00	
Go-ICP	*x*	−10.71	−10.31	−15.13	−3.46	−10.83	10.0	720 s
	*y*	−3.15	−4.19	−3.13	0.62	−1.27	2.47	
	*z*	2.07	3.27	1.34	3.14	4.05	2.77	
CPD-ICP (rigid)	*x*	6.19	5.09	−0.10	7.51	1.19	4.02	840 s
	*y*	−2.50	−3.80	−3.72	−0.97	−1.30	2.46	
	*z*	−10.98	−9.90	−11.36	−9.69	−9.44	10.2	
CPD-ICP (affine)	*x*	3.58	2.46	−3.04	4.23	−1.68	3.00	6 s
	*y*	−0.74	−1.85	−2.14	1.08	1.28	1.42	
	*z*	1.28	1.83	−0.72	0.88	3.43	1.63	
